# Lipoprotein Lipase (LPL) Gene Mutation in a Girl With Diabetic Ketoacidosis, Acute Pancreatitis, and Hypertriglyceridemia

**DOI:** 10.7759/cureus.87126

**Published:** 2025-07-01

**Authors:** Duygu Düzcan Kilimci, Alkan Bal, Ferda Ozkinay, Betül Ersoy

**Affiliations:** 1 Departmana of Pediatric Endocrinology, Manisa Celal Bayar University, Faculty of Medicine, Manisa, TUR; 2 Department of Pediatric Emergency, Manisa Celal Bayar University, Faculty of Medicine, Manisa, TUR; 3 Division of Pediatric Genetics, Ege University, Faculty of Medicine, İzmir, TUR; 4 Department of Pediatric Endocrinology, Manisa Celal Bayar University, Faculty of Medicine, Manisa, TUR

**Keywords:** acute pancreatitis, apolipoproteins, children, hypertriglyceridemia, lipoprotein lipase gene

## Abstract

The combination of acute pancreatitis (AP), severe hypertriglyceridemia (HTG), and diabetic ketoacidosis (DKA) poses a life-threatening triad. Although DKA is a frequent complication in children, this triad is rare. We report a 10-year-old girl with type 1 diabetes mellitus (T1DM) for 10 months, who presented with DKA, severe HTG, and AP. Her serum was lipemic. She had HTG (1733 mg/dl, 19.5 mmol/L; reference range, 90-129 mg/dl, 1,016-1,456 mmol/L) and severe abdominal pain that did not improve despite treatment for ketoacidosis. She had high lipase levels (1581 U/L, reference range 28-100 U/L), and pancreatitis was detected on abdominal tomography. She recovered with a combination of hydration and insulin therapy. A heterozygous p.N318S (c.953A>G) variant was detected in her lipoprotein lipase (LPL) gene. Her apolipoprotein B (ApoB) was elevated at 1.44 g/L (reference range, 0.6-1.17 g/L, 60-117 mg/dl). It is well established that both the likely pathogenic LPL variants and high ApoB concentrations contribute to an increased risk of cardiovascular complications. Therefore, it is recommended to evaluate for a pathogenic variant in the LPL gene in children with T1DM who do not have dyslipidemia but exhibit the rare triad of AP, HTG, and DKA.

## Introduction

The association of diabetic ketoacidosis (DKA), severe hypertriglyceridemia (HTG), and acute pancreatitis (AP) has been reported in only a small number of children and adolescents with type 1 diabetes mellitus (T1DM). The incidence of AP and DKA can be as high as 15% in adults and 2% in children. Hypertriglyceridemia is observed in approximately 8% of adults with DKA. To our knowledge, this is the first pediatric case of the DKA/HTG/AP triad with an identified LPL gene mutation. This triad, which is uncommon during childhood, can lead to severe consequences [1-8]. The mortality rate in DKA is increased in the presence of organ failure [7]. The presence of HTG in the triad can be attributed to insulin deficiency. Insulin deficiency reduces the activity of lipoprotein lipase, which hinders the clearance of very low-density lipoproteins (VLDL) and chylomicrons from plasma, resulting in HTG [3,5]. However, HTG and AP do not occur in most children who develop DKA as a result of insulin deficiency. Therefore, there may be genetic factors contributing to the development of HTG as well.

One of the rare genetic causes of moderate or severe HTG is lipoprotein lipase (LPL) deficiency resulting from a mutation in the LPL gene [9]. To date, no cases have been previously reported that exhibit the triad of DKA/HTG/AP along with an LPL gene mutation. Here, we described a girl diagnosed with T1DM for 10 months who was admitted with the rare triad of DKA, AP, and HTG during treatment. Additionally, a potentially pathogenic LPL variant was detected in her genetic analysis.

This article was previously presented as a poster at the 2023 BSPED 50th Annual Meeting of the British Society for Paediatric Endocrinology and Diabetes on November 8-10, 2023.

## Case presentation

A 10-year-old girl, who had been treated for T1DM for 10 months, presented with nausea, vomiting, and abdominal pain radiating to the back over the past two days. Her regular treatment regimen consisted of three daily doses of insulin lispro and once-daily administration of insulin glargine. However, it was discovered that the patient had not been using insulin doses regularly for the past three months. At the time of diagnosis, her haemoglobin A1c (HbA1c) levels were 15.5% (145.92 mmol/mol, % reference range 5.7-6.4%), which reduced to 10.8% (95.0 mmol/mol) after 3.5 months of treatment.

On admission, she was severely dehydrated, the Glasgow Coma Scale was 13, her oropharynx mucous membrane was dry, and there was tenderness in the epigastric region. Vital signs were recorded as follows: body temperature of 36.7°C, pulse rate of 90/min, respiratory rate of 44/min with Kussmaul pattern, and blood pressure of 100/70 mmHg. Initial blood glucose was 450 mg/dL (24.9 mmol/L, reference range: 60-100 mg/dL), and venous blood gas analysis was suggestive of metabolic acidosis with a pH of 6.8 (reference range: 7.37-7.45), bicarbonate (HCO3) of 4.6 mmol/L (reference range: 22-26 mmol/L), and pCO2 level of 19.6 mmHg (reference range: 35-45 mmHg). The initial biochemical analysis could not be performed due to the lipemic serum. Serum electrolyte levels were found to be very low, with sodium measured at 110 mmol/L (reference range: 135-145 mmol/L) and potassium at 3 mmol/L (reference range: 3.5-5.5 mmol/L). It was determined that these low levels were likely a result of pronounced lipemia. Initially, the patient’s serum amylase and lipase levels were 434 U/L (reference range: 28-100 U/L) and 269 U/L (reference range: 7-39 U/L), respectively. After eight hours, these levels increased to 1877 U/L and 1581 U/L, respectively. No pathological findings were detected on abdominal ultrasound. Serum triglyceride level (TG) was found to be high at 1733 mg/dL (19.5 mmol/L), as shown in Table 1 (reference range: 90-129 mg/dL, 1.0-1.4 mmol/L in 10-19 years).

**Table 1:** Pancreatic enzymes and lipids over time Serum amylase, lipase, and triglyceride levels during hospitalization

	1st day	2nd day	3rd day	4th day	6th day	13th day	Reference range
Amilase	434 U/L	1877 U/L	1508 U/L	357U/L	181 U/L	234 U/L	28-100 U/L
Lipase	269 U/L	1581 U/L	617 U/L	105 U/L	81 U/L	67 U/L	7-39 U/L
Triglyceride	-	-	1733 mg/dl	732 mg/dl	112 mg/dl	167 mg/dl	90-129 mg/dl

However, this measurement was obtained two days after the patient received initial treatment since she was hospitalized over the weekend and the serum lipid level could not be measured in our laboratory on the weekend. Therefore, it was hypothesized that the initial triglyceride level would have been even higher, although it was not measured. Her total cholesterol (TC) was 617 mg/dL (15.9576 mmol/L) (reference range: 190-224 mg/dL, 4.9-5.7 mmol/L), and VLDL was 347 mg/dL (8.9 mmol/L) (reference range: 10-40 mg/dL, 0.1-1.0 mmol/L). At the time of initial diabetes diagnosis, her lipid levels were normal.

Intravenous fluids and insulin infusion were initiated at a rate of 0.1 U/kg/hour after diagnosing DKA. In addition, a proton pump inhibitor was added to her treatment.

Serum creatinine increased on the second day of hospitalization. The patient had a fever. Contrast-enhanced abdominal tomography revealed a bulky pancreas with peripancreatic fluid enhancement, compatible with acute pancreatitis.

**Figure 1:**
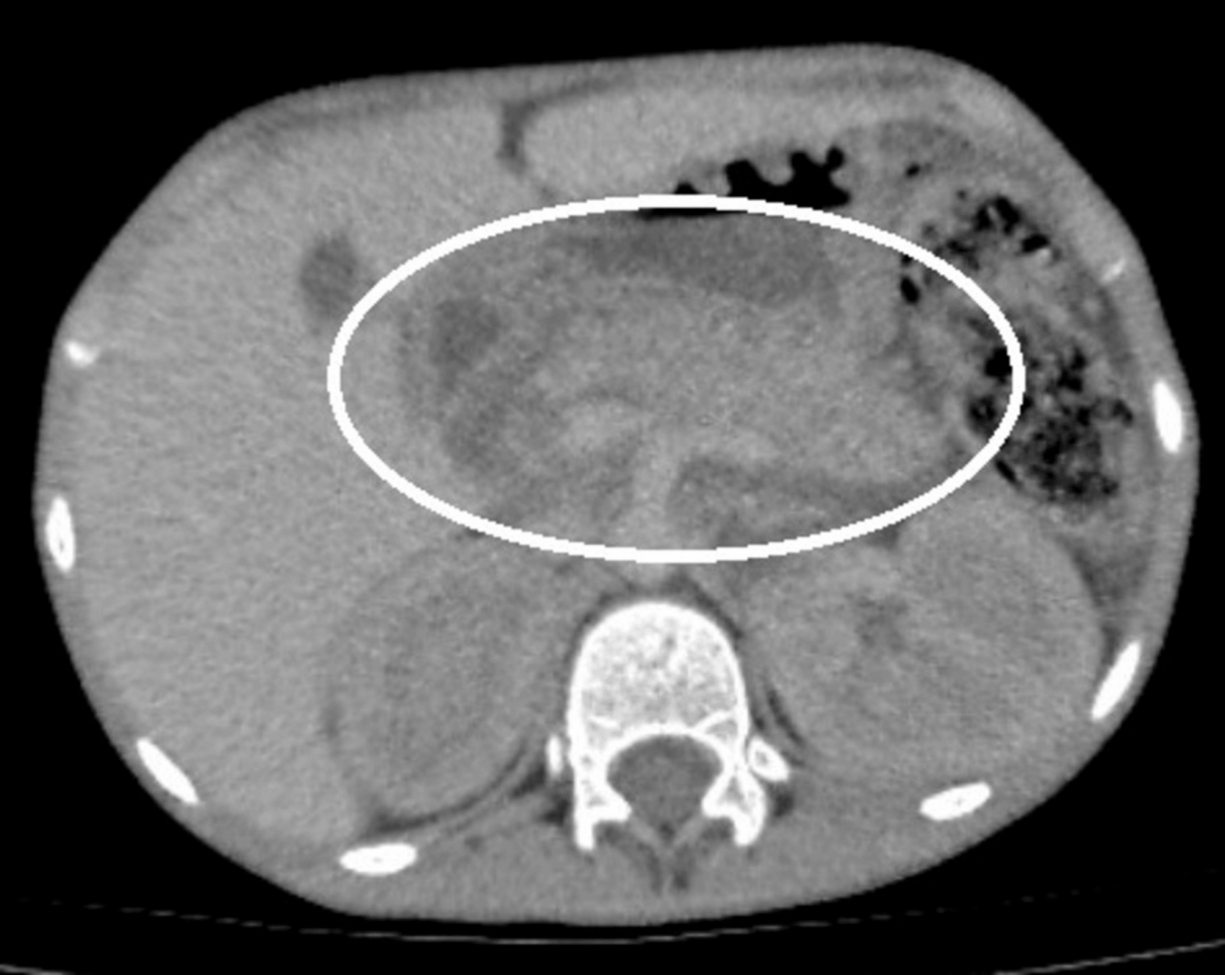
Contrast-enhanced abdominal CT showing pancreatic enlargement with peripancreatic fluid collections White circle showing Balthazar grade E pancreatitis.

According to the Balthazar classification, the patient was diagnosed with stage E acute pancreatitis with two poorly defined peripancreatic fluid collections on abdominal tomography (Figure 1). 

One day after initiating the insulin infusion, the patient’s triglyceride level fell to 732 mg/dL. Therefore, plasmapheresis was not required. On the fourth day, the lipase concentration decreased. On the fifth day, acute renal failure occurred. Blood gas analysis showed a pH of 7.33 and HCO3 of 11.1 mmol/L. With the administration of fluid and bicarbonate treatment, creatinine levels returned to normal after 10 days (Table 2).

**Table 2:** Blood investigations

	1st day	2^nd^ day	3rd day	4th day	6th day	7th day	8th day	13th day	18th day	Reference range
Urea	-	26	36	62	64	77.3	84	50	48	20-50 mg/dl
Creatinine	0.39	1.24	1.61	2.15	2.3	2.51	2.12	1.21	0.87	0.7-1.0 mg/dl
pH	6.82	7.26	7.34	7.23	7.36	7.3	7.37	-	-	7.35-7.45
HCO₃	4.6	11.8	11.5	9.92	11.68	15.19	20.1	-	-	16-22 mmol/lt

Due to a significant increase in HTG levels, the patient underwent genetic testing for LPL (lipoprotein lipase gene) mutations. The test revealed that the patient had a heterozygous pathogenic variant in the LPL gene, p.Asn318Ser (c.953A>G). After discharge, the patient was admitted to the emergency department for ketoacidosis on four occasions within a year. During these attacks, HTG was not present. At the moment of this case report, she has had no attack of ketoacidosis over a duration of about two years. Her apolipoprotein (Apo) concentrations were also evaluated. Apo A was 1.20 g/L (reference range: 1.08-2.25 g/L, 108-225 mg/dL), and Apo B was 1.44 g/L (reference range: 0.6-1.17 g/L, 60-117 mg/dL).

## Discussion

Diagnostic challenges

This is the first case demonstrating the presence of an LPL gene mutation in a child with T1DM who presented with the triad of DKA, HTG, and AP. Our patient initially presented with severe abdominal pain, lumbar pain, and vomiting. Abdominal pain and vomiting are common manifestations of both DKA and acute pancreatitis. Therefore, these signs may be attributed to DKA, but they can actually mask the presence of acute pancreatitis. Although the co-existence of DKA and acute pancreatitis is rare, it should be considered in the presence of severe abdominal pain [10].

In addition to clinical findings, our patient had elevated serum lipase and amylase levels. It has been documented that children with DKA without AP may experience elevated serum amylase and lipase levels due to hypoperfusion. Pancreatic enzymes are elevated 12-24 hours after the initiation of ketoacidosis therapy. Pancreatic enzyme elevation is not always associated with abdominal pain [11]. However, in cases that have reported AP coexistent with DKA, the elevation of pancreatic enzyme levels is 10-15 times greater than normal [3,6-8,11], as in our case.

Adults with the DKA/HTG/AP triad have an increased rate of mortality due to multi-organ failure. Acute renal failure occurred in the presented case. HTG plays a crucial role in this triad and is believed to be the underlying cause of acute pancreatitis, as supported by previous studies [1,3,5,7]. Our patient had lipemia, which led to a false decrease in serum sodium and other electrolyte levels (pseudo hyponatremia) on admission. In the present case, lipids were normal both before and after the presentation with the DKA/HTG/AP triad, as in the previously reported pediatric patients with this triad [1,3]. The improvement of most cases with insulin and hydration therapy, as occurred in the present case, supports the role of insulin deficiency in pathogenesis [1,3-6]. However, alternative treatment approaches have also been reported, including the use of plasmapheresis [12] and fenofibrate [6,12].

Genetic analysis

A heterozygous variant, p.Asn318Ser (NM_000237.3: c.953A>G), in the 6th exon of the LPL gene, was found in the presented patient. As ClinVar classifications, the variant is conflicting interpretations of pathogenicity (ClinGen:CA251887) [14]. This variant is found in approximately 1-7% of individuals in Caucasian populations [13] and is more prevalent among individuals with dyslipidemia compared to those without the condition. Furthermore, a notable interaction between p.Asn318Ser and insulin resistance was observed in normoglycemic individuals, suggesting that dyslipidemia is more severe in p.Asn318Ser carriers with decreased insulin sensitivity. Additionally, this variant has been linked to an increased risk of ischemic heart disease in women [13]. However, several studies have reported no association between this variant and dyslipidemia or cardiovascular disorders. As a result, the pathogenicity of this variant remains controversial [13]. With the exception of the reported episode, the presented patient did not have dyslipidemia, which is consistent with the majority of individuals carrying the p.Asn318Ser. However, in children and adolescents with T1DM who carry this variant, when environmental stress such as insulin deficiency occurs, HTG and the associated clinical picture may occur, as in the present case.

Clinical implications 

The combination of acute pancreatitis (AP), severe hypertriglyceridemia (HTG), and diabetic ketoacidosis (DKA) poses a life-threatening triad. Although DKA is a frequent complication in children, this triad is rare. We report a 10-year-old girl with type 1 diabetes mellitus (T1DM) for 10 months, who presented with DKA, severe HTG, and AP. She had HTG (1733 mg/dl, 19.5 mmol/L; reference range, 90-129 mg/dl, 1.016-1.456 mmol/L) and severe abdominal pain that did not improve despite treatment for ketoacidosis. She had high lipase levels (1581 U/L, reference range: 28-100 U/L), and pancreatitis was detected on abdominal tomography. She recovered with a combination of hydration and insulin therapy. A heterozygous p.Asn318Ser (c.953A>G) variant was detected in her lipoprotein lipase (LPL) gene. Her apolipoprotein B (ApoB) was elevated at 1.44 g/L (reference range, 0.6-1.17 g/L, 60-117 mg/dL). It is well established that both the likely pathogenic LPL variants and high ApoB concentrations contribute to an increased risk of cardiovascular complications. Therefore, it is recommended to evaluate for a pathogenic variant in the LPL gene in children with T1DM who do not have dyslipidemia but exhibit the rare triad of AP, HTG, and DKA.

## Conclusions

In conclusion, the presence of LPL gene mutation should be considered in the presence of type 1 diabetic children and adolescents with no previous dyslipidemia who present with rare severe hypertriglyceridemia, diabetic ketoacidosis, and acute pancreatitis. Detection of these mutations may also be important in predicting long-term cardiovascular risks and may enable more attention to cardiovascular risk during patient follow-up. The LPL variant (p.Asn318Ser) significantly influences the biochemical phenotype and risk for cardiovascular disease. Determination of apolipoprotein concentration may be useful in predicting LPL gene mutation in children with T1DM who have the triad.
